# Low-Bone-Mass Phenotype of Deficient Mice for the Cluster of Differentiation 36 (CD36)

**DOI:** 10.1371/journal.pone.0077701

**Published:** 2013-10-25

**Authors:** Olha Kevorkova, Corine Martineau, Louise Martin-Falstrault, Jaime Sanchez-Dardon, Louise Brissette, Robert Moreau

**Affiliations:** 1 Laboratoire du Métabolisme osseux, Centre BioMed, Département des sciences biologiques, Université du Québec à Montréal, Montréal, Québec, Canada; 2 Laboratoire du Métabolisme des Lipoprotéines, Centre BioMed, Département des sciences biologiques, Université du Québec à Montréal, Montréal, Québec, Canada; Inserm U606 and University Paris Diderot, France

## Abstract

Bone tissue is continuously remodeled by bone cells and maintenance of its mass relies on the balance between the processes of resorption and formation. We have reported the expression of numerous scavenger receptors, namely scavenger receptor (SR) class B type I and II (SR-BI and SR-BII), and CD36, in bone-forming osteoblasts but their physiological roles in bone metabolism are still unknown. To unravel the role of CD36 in bone metabolism, we determined the bone phenotype of CD36 knockout (CD36KO) mice and characterized the cell functions of osteoblasts lacking CD36. Weights of CD36KO mice were significantly lower than corresponding wild-type (WT) mice, yet no significant difference was found in femoral nor tibial length between CD36KO and WT mice. Analysis of bone architecture by micro-computed tomography revealed a low bone mass phenotype in CD36KO mice of both genders. Femoral trabecular bone from 1 to 6 month-old CD36KO mice showed lower bone volume, higher trabecular separation and reduced trabeculae number compared to WT mice; similar alterations were noticed for lumbar vertebrae. Plasma levels of osteocalcin (OCN) and N-terminal propeptide of type I procollagen (PINP), two known markers of bone formation, were significantly lower in CD36KO mice than in WT mice, whereas plasma levels of bone resorption markers were similar. Accordingly, histology highlighted lower osteoblast perimeter and reduced bone formation rate. *In vitro* functional characterization of bone marrow stromal cells and osteoblasts isolated from CD36KO mice showed reduced cell culture expansion and survival, lower gene expression of osteoblastic Runt-related transcription factor 2 (Runx2) and osterix (Osx), as well as bone sialoprotein (BSP) and osteocalcin (OCN). Our results indicate that CD36 is mandatory for adequate bone metabolism, playing a role in osteoblast functions ensuring adequate bone formation.

## Introduction

Bone is a dynamic tissue that undergoes continual remodeling through the coordinated processes of bone resorption and formation. The equilibrium of both processes ensures the preservation of skeleton structural integrity and mineral homeostasis of the organism [1]. Osteoclasts and osteoblasts are specialized cells which respectively break down old bone tissue and promote the formation of a novel bone matrix. Osteoblasts derive from bone marrow mesenchymal precursor cells that sequentially differentiate into proliferating preosteoblasts, bone matrix-producing osteoblasts, and eventually into osteocytes embedded in the bone matrix under the control of specific transcription factors [2]. Differentiated osteoblasts synthesize and secrete type I collagen, the main bone matrix protein, and also regulate bone mineralization by expressing alkaline phosphatase (ALP) and osteocalcin (OCN). In addition, these cells continuously orchestrate bone remodeling by regulating the activation and differentiation of cells from the monocyte/macrophage lineage into osteoclasts [1], required for the resorption process. Any imbalance between the functions of resorptive and formative cell populations that leads to excessive bone resorption or inadequate formation response results in loss of bone density, lower bone mass and increased risk of bone fractures [3] which are the main diagnostic criteria of osteoporosis [4].

Several etiologic factors have been identified for the development of osteoporosis such as hormonal state particularly low oestrogen level and hyperparathyroidism, exposure to certain medications (such as glucocorticoids), calcium deficiency due to low dietary calcium intake or impaired intestinal absorption as well as vitamin D deficiency, compromised antioxidant conditions, hematologic disorders, gastrointestinal and metabolic diseases [3], [5], [6]. A number of recent clinical and experimental studies have linked disorders of the lipoprotein metabolism and atherosclerosis with the development of osteoporosis [7], [8] suggesting that both pathologies share contributory factors. Comprehension of the lipoprotein metabolism has beneficiated from the study of lipoprotein receptors such as SR superfamily and specifically SR class A (SRA), and class B (SR-BI and CD36) [9]. We have recently reported the expression of SR-BI, SR-BII, and CD36 in osteoblasts [10], however their physiological roles in bone metabolism are still unknown.

CD36 is an integral membrane glycoprotein and its expression has been demonstrated in platelets, monocytes/macrophages, megakaryocytes, microvascular endothelial cells, adipocytes, hepatocytes, cardiac and skeletal myocytes, and bone cells [9], [10], [11]. CD36 binds a variety of extracellular ligands and thus, this receptor has been implicated in a broad range of biological functions. In endothelial cells, CD36 has been implicated in angiostatic response and apoptosis through binding of thrombospondin-I [12]. CD36 has been involved in innate and adaptive immunity due to its ability to recognize lipid and lipoprotein components of bacterial cell wall such as lipoteichoic acid and lipopolysaccharides [13]. CD36 has been associated with adherence to microvascular endothelial cells and phagocytosis of *Plasmodium falciparum*-parasitized erythrocytes by macrophages [14]. In relation to lipid and lipoprotein metabolism, CD36 promotes long-chain fatty acid transport and intracellular lipid accumulation [15]. Moreover, low density lipoproteins (LDL) when oxidized (oxLDL) enhance CD36 expression in macrophages which leads to endocytosis of oxLDL, formation of cholesterol loaded foam cells and initiation of atherosclerotic lesions [16]. Additionally, CD36-mediated oxLDL binding by macrophages induces the production of inflammatory cytokines, which generates local inflammation in atherosclerotic plaque [9]. Moreover, atherosclerotic lesions in CD36 deficient (KO) mice are reduced when compared to apolipoprotein E/CD36 double KO, associating proatherogenic properties to CD36 [17]. Furthermore, plasma levels of cholesterol, free fatty acid and triacylglycerol [18] increase in CD36KO mice and such dysfunctions of lipid metabolism have been associated to impaired lipid uptake and decreased lipolysis [19], [20]. CD36 has been implicated in platelet aggregation and thrombus formation in dyslipidemic state [21], [22], [23], as CD36KO mice show reduction of thrombotic vessels occlusion and aggregation of platelets in hyperlipidemic state [22]. A recent study using CD36KO mice demonstrated the involvement of CD36 in the antiangiogenic response related to the pathophysiology of choroidal involution and corneal neovascularisation [24], [25]. Cornea of CD36KO mice showed increased age-dependent neovascularisation with subsequent enhancement of inflammation and expression of angiogenic factors [24]. In contrast, choroid of CD36KO mice was characterized by increased avascular area with severe thinning of choroid and diminished expression of angiogenic factors [25].

Given the implication of CD36 in a broad range of physiological functions and its expression by osteoblasts, the current study was undertaken to unravel the role of CD36 in bone metabolism by determining the bone phenotype of CD36KO mice and characterizing the cell functions of osteoblasts lacking CD36.

## Materials and Methods

### Animals

CD36KO mice were obtained from Dr Maria Febbraio (Cleveland, Ohio) and were backcrossed at least 7 times to wild-type (WT) C57BL/6J mice purchased from Charles River (Boston, MA, USA). One to 6 month-old mice were used and the animals were provided with a standard mouse chow diet and drinking water. CD36 genotyping was done by PCR as described previously [26] using specific primers for the targeted allele (5′-CAGCTCATACATTGCTGTTTATGCATG and 3′-CGCTTCCTCGTGCTTTACGGTATC). This study was conducted according to protocols approved by the Animal Care and Use Committee of Université du Québec à Montréal.

### Plasma analysis

Blood was collected into heparin collection tubes (68 USP, BD Bioscience) by cardiac puncture of anaesthetised mice, prior to their euthanasia. Blood was centrifuged for 30 min at 2000g and 4°C, and plasma was recovered and stored at −80°C until analysis. Plasma levels of glucose, calcium, phosphate and ALP activity were determined using QuantiChrom Assay Kits (BioAssay System, Hayward, CA). Plasma concentrations of total cholesterol, high density lipoprotein (HDL)- and LDL-cholesterol were measured using EnzyChrom AF HDL and LDL/VLDL Assay Kit (BioAssay System, Hayward, CA) according to the manufacturer's instructions. Plasma levels of tartrate-resistante acid phosphatase (TRAP) isoform 5b, N-terminal propeptide of type I procollagen (PINP) and C-terminal telopeptide of type I collagen (CTX) were measured by EIA assays (IDS Inc, Fountain Hills, AZ). Osteocalcin detection in plasma was done using a mouse EIA kit (Biomedical Technologies Inc, Stoughton, MA) according to the manufacturer's instructions.

### Documentation of bone architecture

MicroCT analyses were performed using a Skyscan 1172c X-ray computed microtomograph (Skyscan, Kontich, Belgium) equipped with an X-ray tube working at 70 kV/100 µA. Femura and lumbar vertebrae (L4–L5) of WT and CD36KO mice were scanned at a 5 µm resolution, a 180° rotation with a 0.5° rotation increment and a 0.5 mm aluminum filter. A stack of 2D X-ray shadow projections was reconstructed to obtain cross-sectional images using NRecon software (Skyscan), and subjected to morphometric analyses using CTAn software (Skyscan). Trabecular parameters were measured at the metaphysis (a total of 300 slices were selected) and cortical parameters were determined at the femoral diaphysis (a total of 100 slices were selected), and 200 slices were chosen either side of the L4–L5 vertebrae. The three-dimensional morphometric parameters of bone microarchitecture were calculated using CTAn (Skyscan) software. The parameters measured included bone volume fraction (bone volume/total volume (BV/TV)), trabecular thickness (Tb.Th), number (Tb.N), and separation (Tb.Sp) for trabecular bone and vertebrae, and BV, cortical thickness (Cort.Th), endocortical perimeter (Ec.Pm) and periosteal perimeter (Ps.Pm) for cortical bone. Three-D renderings were generated from these volumes of interest using CTvol software (Skyscan).

### Bone histochemistry

Twenty mg of calcein (Sigma-Aldrich, Oakville, Ont., Canada) per kg body weight were injected intraperitoneally to 4-week-old mice on days 9 and 2 prior to euthanasia (day 0). The femura were dissected free of soft tissue, fixed for 16 h in 4% paraformaldehyde (PF) at 4°C and rinsed in phosphate buffer saline (PBS; 1 mM CaCl_2_, 2.7 mM KCl, 1.4 mM KH_2_PO_4_, 0.8 mM MgCl_2_.6H_2_O, 137 mM NaCl, 10 mM Na_2_HPO_4_, pH 7.4). The femora were embedded in a mixture of polymethylmethacrylate (PMMA) as described by Erben [27], 6 µm sections were cut with a Thermofisher rotary HM 360 microtome. Staining for ALP (Millipore, Billerica, MA, USA) and TRAP activity (K Assay, Dako, Burlington, Ont., Canada) was carried out at 37°C in a Coplin jar placed in a moist chamber as described previously [28]. Bone sections were visualized with an inverted phase contrast microscope (Nikon Eclipse Ti, Mississauga, Ont, Canada) and analyzed with ImageJ software to determine relative ALP-positive osteoblast perimeter (ObPm) and number of TRAP positive osteoclasts (#Oc/mm). Calcein labeling was visualized with a Nikon FN1 Eclipse inverted fluorescence microscope and used to evaluate mineralizing surface (MS/BS), mineral apposition rate (MAR) and bone formation rate (BFR).

### Primary cultures of osteoblasts

For primary cultures of bone marrow mesenchymal stromal cells (MSC) and osteoblasts, 4–8 week old animals were euthanatized and femora and tibiae from WT and CD36KO mice were collected and carefully cleaned from adherent tissues. Bones were broken in half and centrifuged 5 min at 2500 rpm for the collection of bone marrow cells. Cell pellets were re-suspended in culture medium, seeded in 100-mm dishes (Sarstedt, Montréal, Québec, Canada), and allowed to adhere for 48 h in α-MEM with phenol (Invitrogen, Burlington, Ontario, Canada) supplemented with 20% fetal bovine serum (FBS; NorthBio, Toronto, Ontario, Canada), L-glutamine (Invitrogen) and penicillin/streptomycin (Invitrogen). Non-adherent cells were discarded and adherent cells were washed with PBS and cultured in supplemented α-MEM with FBS until confluent. The resulting MSC cultures were lifted by incubation in 0.05% trypsin-0.02% EDTA solution (Invitrogen) and cell phenotype was analysed by flow cytometer (Becton-Dickinson). The cell suspensions were washed in PBS and a total of 1×10^5^ cells were double-stained for 15 min at room temperature with monoclonal antibodies against mouse CD105 conjugated to phycoerythrin (PE) and mouse CD73 conjugated to fluorescein isothiocyanate (FITC) (BioLegend, San Diego, CA). Afterwards, fluorescence was measured in 10,000 cells per sample by flow cytometry and analysis was performed using Cell-Quest 3.1 software (Becton-Dickinson). The mouse mesenchymal cell line C3H10T1/2 (ATCC, cultured in DMEM medium (Sigma)) was used as positive control for MSC antigens. Remaining cells were seeded in appropriate plates for subsequent experiments. For primary cultures of osteoblasts, remaining bones were further chopped into fine pieces with a scalpel. Next, the bone fragments were further washed twice PBS and incubated three times at 37°C with 1 mg/mL of collagenase type I (Sigma, Oakville, Ontario, Canada) in α-MEM without FBS for 20, 20, and 40 min. After, the bone fragments were washed twice with PBS and transferred into 100-mm culture dishes containing α-MEM supplemented with 10% FBS. Digested bone fragments were cultured until cell outgrowth and typically reached confluence within 14–21 days in culture. At confluence, cells were sub-cultured and seeded in appropriate plates for subsequent experiments.

### Alkaline phosphatase activity

For measurement of ALP activity, cells from WT and CD36KO mice were seeded in 24-well plates and cultured for 7 days. Thereafter, cells monolayers were washed three times with PBS then solubilised in ice-cold assay buffer (100 mM glycine, 1 mM MgCl_2_, 0.5% Triton X-100, pH 10.5) for ALP activity determination by conversion of *para*-nitrophenylphosphate (*p*-NPP, Sigma) into *para*-nitrophenolate (*p*-NP) as described previously [29]. Briefly, 75 µL of lysate was mixed with 75 µL of freshly prepared colorimetric substrate *p*-NPP (12.5 mM) solubilized in the assay buffer. The enzymatic reaction was conducted for 1 h at 37°C and was stopped by adding 100 µL of NaOH 1 M. Absorbance of the yellow product p-NP was determined spectrophotometrically at 410 nm. Protein concentration was quantified by MicroBCA protein assay (Pierce, Rockford, IL, USA) using bovine serum albumin as standard. ALP activity was then expressed as p-NP produced in nmol/h/mg of cellular protein.

### MTT activity

For cell expansion experiments, cells from WT and CD36KO mice were seeded in 96-well plates and cultured for 7 days in FBS-supplemented α-MEM. MTT activity was determined by microtiter tetrazolium 3-(4,5-dimethylthiazol-2-yl)-2,5-diphenyltetrasodium bromide (MTT) reduction assays at day 1, 4 and 7 post-seeding. Briefly, 2 h before the end of treatment, media were replaced with media containing 0.5 mg/mL MTT (Sigma). At the end of the incubation, media were withdrawn and formazan crystals generated by the cellular reduction activity were dissolved in dimetylsulfoxide. Absorbance was measured at 575 nm, and data are expressed as the ratio of absorbance *vs* values for WT cells of day 1. To corroborate MTT assay with cell culture expansion, cells in 12-well plates were trypsinized after culture period of 7 to 11 days, suspended in PBS and counted with a haemocytometer. For cell survival experiments, cells were cultured 7 days and further cultured for an additional 14-day period in differentiating medium (α-MEM containing 10% FBS and supplemented with 10 mM β-glycerophosphate (Sigma), and 50 µg/mL ascorbic acid (Sigma)). Cell viability was determined by MTT assays as described above and expressed as the ratio of absorbance *vs* values for WT cells of day 0, or by the determination of cellular protein by MicroBCA assays.

### PCR amplification

For gene expression analysis, the bone marrow MSC from WT and CD36KO mice were seeded in 60-mm culture dishes and incubated for 7 days. Total RNA was extracted from cells using TriZol (Invitrogen) according to the manufacturer's instructions. Reverse transcription (RT) reactions were carried out with Omniscript RT kit (Qiagen, Mississauga, Ontario, Canada) using hexamers. PCR amplifications for semi-quantitative analysis were conducted with *Taq* PCR core kit (Qiagen) using specific primer sets for OCN (F: 5′-CAAGTCCCACACAGCAGCTT-3′, R: 5′AAAGCCGAGCTGCCAGAGTT-3′), BSP (F: 5′-ACTCCAACTGCCCAAGAAGG-3′, R: 5′-CTGTGGTTCCTTCTGCACCT-3′), Osx (F: 5′-TTCGCATCTGAAAGCCCACT-3′, R: 5′-TGCGCTGATGTTTGCTCAAG-3′) and collagen type I alpha 1 (Col1α1; (F 5′-ACTTCAGCTTCCTGCCTCAG-3′, R 5′-GCTTCTTTTCCTTGGGGTTC-3′). GAPDH was used as a reference gene for normalization. Amplifications were carried out for 40 cycles of 1 min at 94°C, 30 s at 58°C and 1 min at 72°C. Amplification products were resolved in 2% agarose gel and were visualized under UV by ethidium bromide staining. Real-time PCR analysis for mouse Runx2 (primers QT00102193 from Qiagen) was performed using the iCycler IQ detection system (Bio-Rad, Hercules, CA, USA) and SYBR Green I (Bio-Rad) as a double-strand DNA–specific binding dye, using β-microglobuline as reference gene (F 5′-TACTCAGCCACCCACCGGCCG-3′, R 5′-GCTCGGCCATACTGGCATGCT-3′)). Each sample was run in triplicate, and fluorescence data were collected at the end of the extension step in every cycle. To ensure specific amplification, a melting curve was calculated for each PCR reaction by increasing the temperature from 60 to 95°C with a temperature increment rate of 0.5°C/10 seconds. Fold induction and expression levels for Runx-2 were calculated using the comparative CT method [i.e., 1/(2ΔC T), where ΔCT is the difference between CT target and CT reference] after normalization to β-microglobuline expression level, and data were analyzed using optical system software Version 3.1 (Bio-Rad).

### Statistical analysis

Data is expressed as mean ± SEM, and the significance of differences between groups has been determined using GrafPadPrism 5.0 Software by analysis of variance. Differences between groups were further evaluated by Student T test or two-way ANOVA with Bonferroni post test. Differences were considered significant at P≤0.05.

## Results

### Body weight, bone length and plasmatic markers

**Figure 1:**
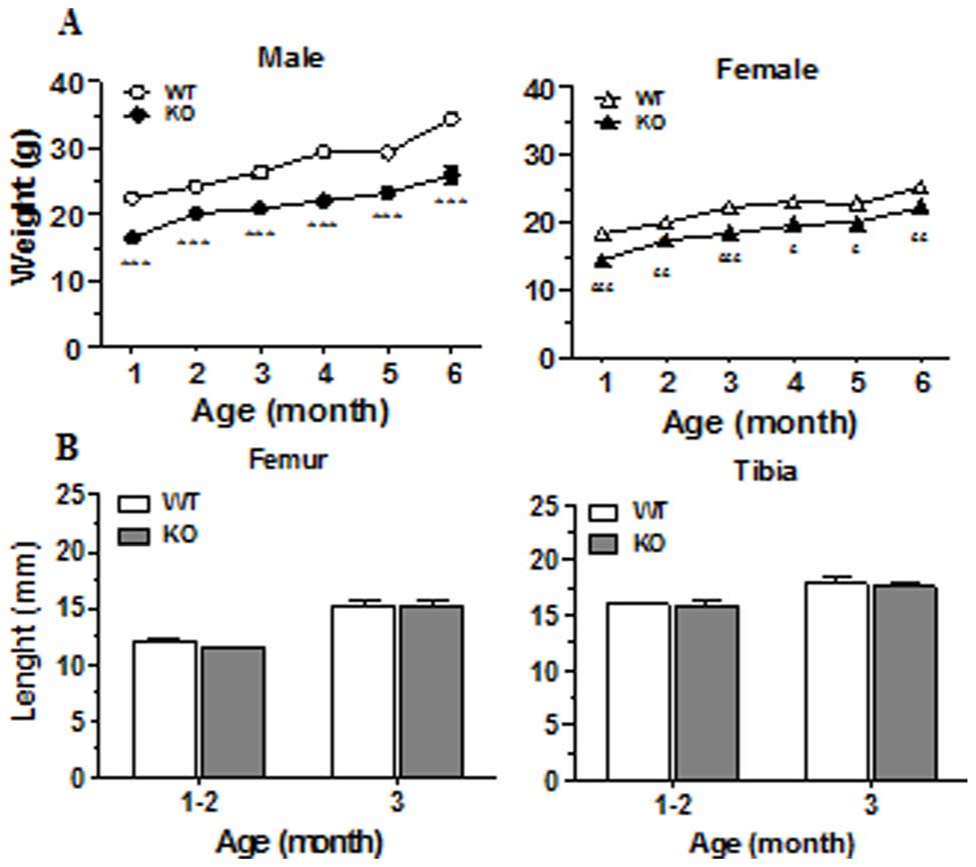
Body weight and bone length of 1 to 6 month-old WT and CD36KO mice. A) Body weight of WT and CD36KO male and female mice from 1 to 6 months of age. Data are expressed as mean ± SEM from 10–37 mice per group of age. Bonferroni post-test: ***P<0.001 compared to WT male; ^£^P<0.05, ^££^P<0.01,^ £££^P<0.001 compared to WT female. B) Femur and tibia lengths for WT and CD36KO mice of 1–3 months old. Data are expressed as mean ± SEM from 11–35 mice per group of age.

We have recently reported the expression of CD36 by osteoblasts [10] and herein, we took advantage of the CD36KO mouse model to investigate the role of CD36 in bone remodeling. First, we determined general morphogenic and plasmatic parameters of CD36KO mice. Weights of CD36KO were significantly lower than WT mice (Fig. 1A), and this difference persisted from 1 to 6 months in male (between 20–25%) and female mice (10–20%). Since this lower weight may reflect a reduction of bone growth, the length of femur and tibia from CD36 and WT mice was compared. There were no significant differences in length of these long bones in sex- and age-matched CD36KO and WT mice (Fig. 1B). Visceral and fat pad adipose tissue was globally reduced in CD36KO mice compared to WT mice (unpublished observations) which may account for the lower weight. Since CD36 has been functionally associated with the metabolism of lipoproteins [17], [18], we measured the plasma levels of total cholesterol and fractions associated with LDL and HDL. The levels of plasma total cholesterol, LDL and HDL fractions were similar in 1 month-old CD36KO and WT mice (Table 1). Moreover, plasma analysis revealed a normal mineral homeostasis for calcium and phosphate, as well as normal levels of glucose and ALP activities in CD36KO mice (Table 2).

**Table 1:** Plasma levels of total cholesterol, HDL cholesterol and LDL cholesterol of WT and CD36KO mice.

Gender	Genotype	Total cholesterol (mg/dL)	HDL cholesterol (mg/dL)	LDL cholesterol (mg/dL)
Male	WT	127.1±6.8	63.6±2.1	63.5±4.7
	KO	96.1±14.6	57.1±2.5	46.7±10.0
Female	WT	98.3±12.6	53.3±9.1	45.1±3.7
	KO	122.4±8.1	70.2±6.7	46.8±3.2

Blood was obtained from 4 weeks old WT (n = 6) and CD36KO (n = 8) mice, then plasma was analyzed as outlined in the Materials and Methods section. Values are means ± SEM.

**Table 2:** Plasma levels of glucose, calcium, phosphate and alkaline phosphatase activity (ALP) of WT and CD36KO mice.

Gender	Genotype	Glucose (mg/dL)	Calcium (mg/dL)	Phosphate (mg/dL)	ALP (U/L/min)
Male	WT	270.4±15.6	17.9±0.9	2.3±0.1	506.0±33.6
	KO	225.6±16.4	18.3±0.9	2.3±0.1	411.8±63.8
Female	WT	256.0±3.4	18.4±1.1	2.2±0.2	545.6±26.6
	KO	280.2±20.9	20.2±0.7	2.1±0.1	493.3±8.9

Blood was obtained from 4 weeks old WT (n = 6) and CD36KO (n = 8) mice, then plasma was analyzed as outlined in the Materials and Methods section. Values are means ± SEM.

### Determination of bone architecture

**Figure 2:**
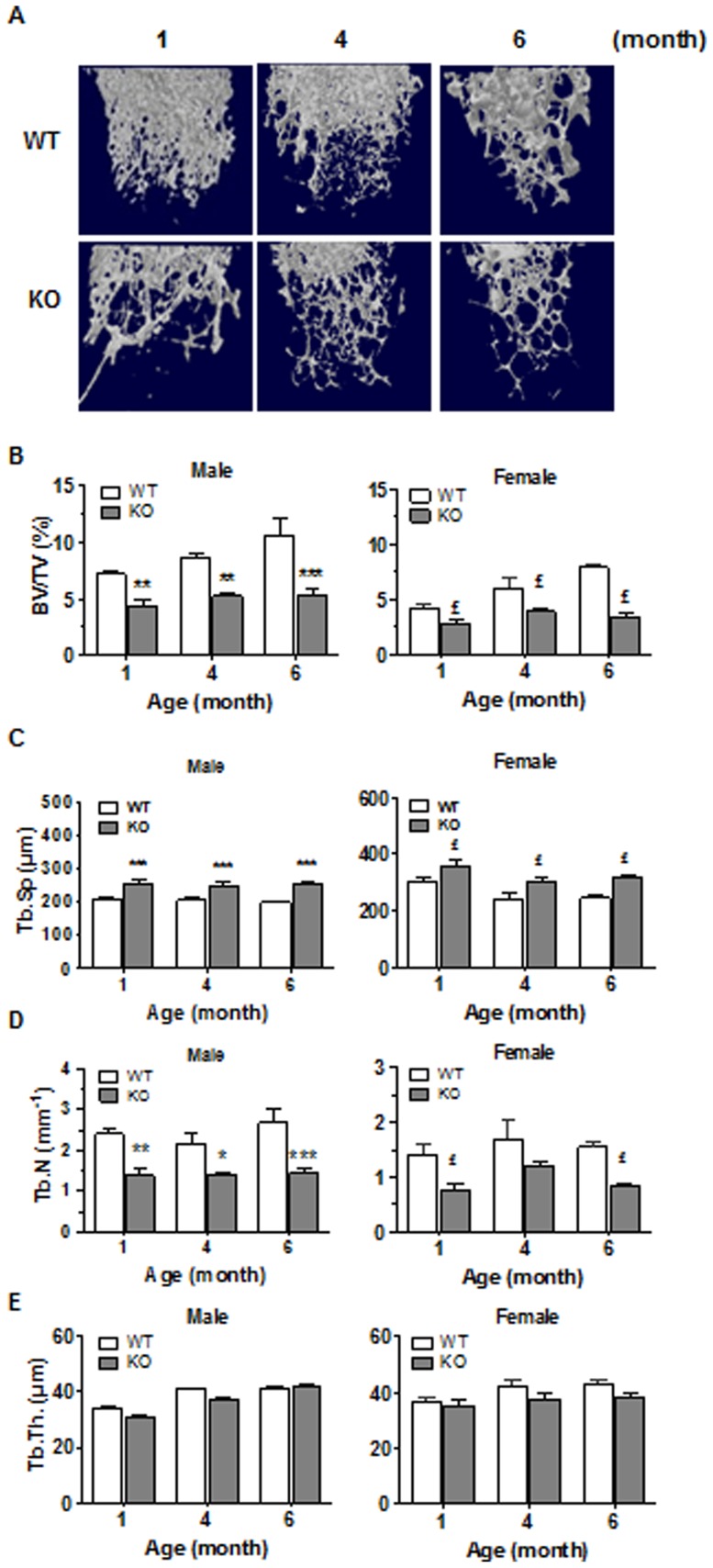
Microarchitecture analysis of trabecular femoral bone of WT and CD36KO mice. (A) Representative 3D reconstructions of femoral trabecular bone from female WT and CD36KO mice. (B) Percent bone volume (BV/TV) of femoral trabecular portion of 1 to 6 month old male and female WT and CD36KO mice. (C) Trabecular spacing (Tb.Sp), (D) Trabeculae number (Tb.N) and (E) trabecular thickness (Tb.Th) of femurs from 1 to 6 months old male and female WT and CD36KO. Values are means ± SEM from 17–22 mice per group of age. Bonferroni post-test: *P<0.05, **P<0.01 and ***P<0.001 compared to WT male; ^£^P<0.05 compared to WT female.

**Figure 3:**
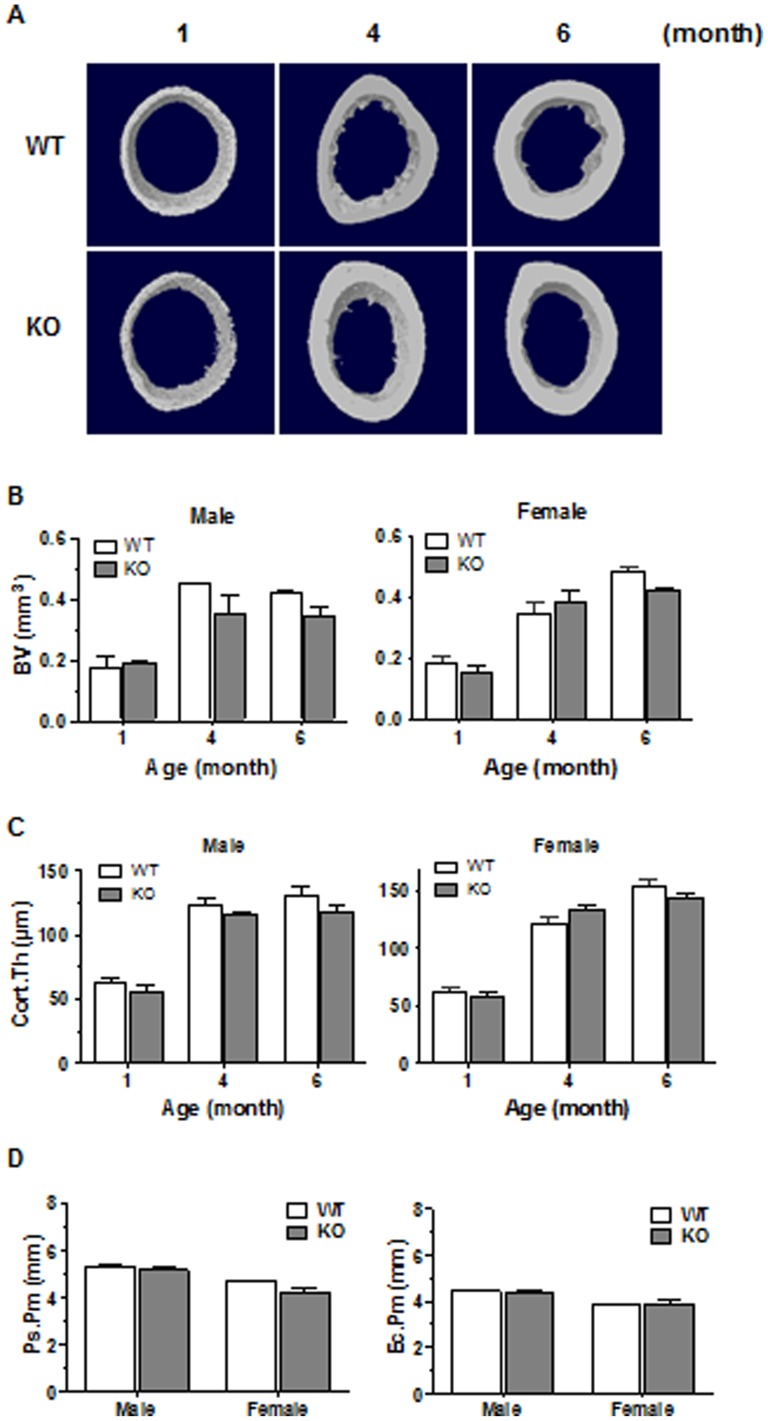
Microarchitecture analysis of cortical femoral bone of WT and CD36KO mice. (A) Representative 3D microCT images of cortical bone at the femoral diaphysis of 1 to 6 month-old female mice. (B) Bone volume (BV), (C) cortical thickness (Cort.Th), (D) periosteal perimeter (Ps.Pm) and endocortical perimeter (Ec.Pm) of cortical portion from femoral diaphysis of 1 month male and female WT and CD36KO mice. Values are means ± SEM from 6–14 mice per group of age.

We next documented whether CD36 deficiency was associated with alterations of bone architecture. Fig. 2 summarizes the visual appreciations (3D reconstructions) and architectural parameters of the femoral trabecular bone of WT and CD36KO mice. In 1 to 6 month-old mice, bone mass was visibly reduced in CD36KO mice (Fig. 2A) and in accordance, the percentages of BV/TV were significantly lower at 1 month of age in male (−48%) and female (−50%) CD36KO compared to WT mice (Fig. 2B). This low bone mass phenotype was also noticed in 3–6 month-old KO individuals. In absence of CD36, Tb.Sp. was increased by an average of 27% in male and of 26% in female mice (Fig. 2C). Also, a significant reduction of Tb.N. was measured in both male (average of 41%) and female (average of 54%) CD36KO mice compared to WT mice (Fig. 2D). However, thickness of trabeculae was not significantly different between WT and CD36KO mice (Fig. 2E). Analysis of femoral cortical bone was also performed, showing a global but not significant drop in femoral cortical bone volume in CD36KO mice indicating that CD36 deficiency does not impact the cortical portion of femura (Fig. 3).

**Figure 4:**
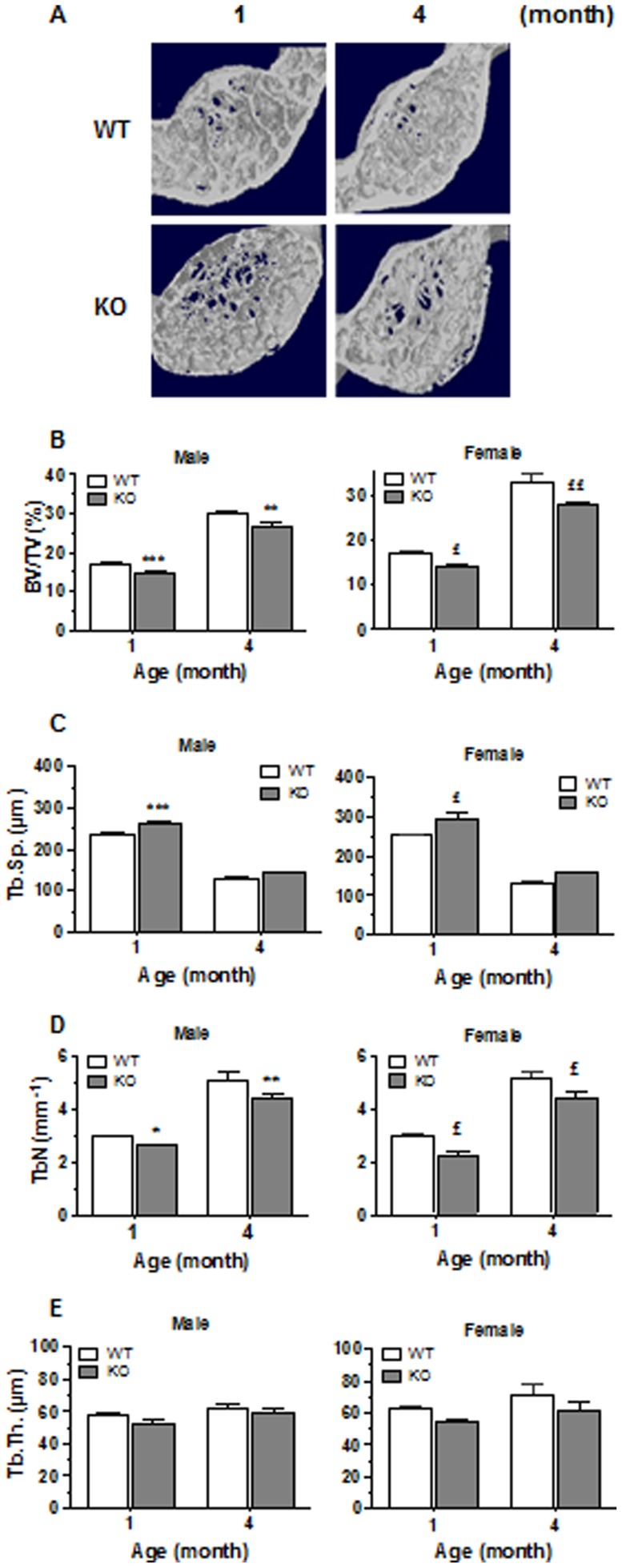
Microarchitecture analysis of vertebrae of WT and CD36KO mice. (A) Representative 3D reconstructions of vertebrae of 1 and 4 month-old female mice. (B) Percent bone volume (BV/TV), (C) trabecular spacing (Tb.Sp.), (D) trabeculae number (Tb.N.) and (E) trabecular thickness (Tb.Th.) of vertebral from male and female WT and CD36KO mice. Values are means ± SEM from 5–18 mice per group of age. Bonferroni post-test: *P<0.05, **P<0.01 and ***P<0.001 compared to WT male; ^£^P<0.05 and ^££^P<0.01 compared to WT female.

To determine whether CD36 deficiency leads to specific alterations of long bone architecture, we also performed analysis of vertebrae. As shown in Fig. 4, the vertebral bone mass was also reduced in CD36KO male (11–15%) and female mice (16–19%). Trabecular separation was enhanced in vertebra for both male (9.4%) and female (13.7%) CD36KO mice (Fig. 4C). The number of trabeculae was significantly lower in CD36KO mice when compared to WT (Fig. 4D), whereas trabecular thickness did not differ (Fig. 4E).

### Plasma levels of bone remodeling markers and bone histochemistry

**Figure 5:**
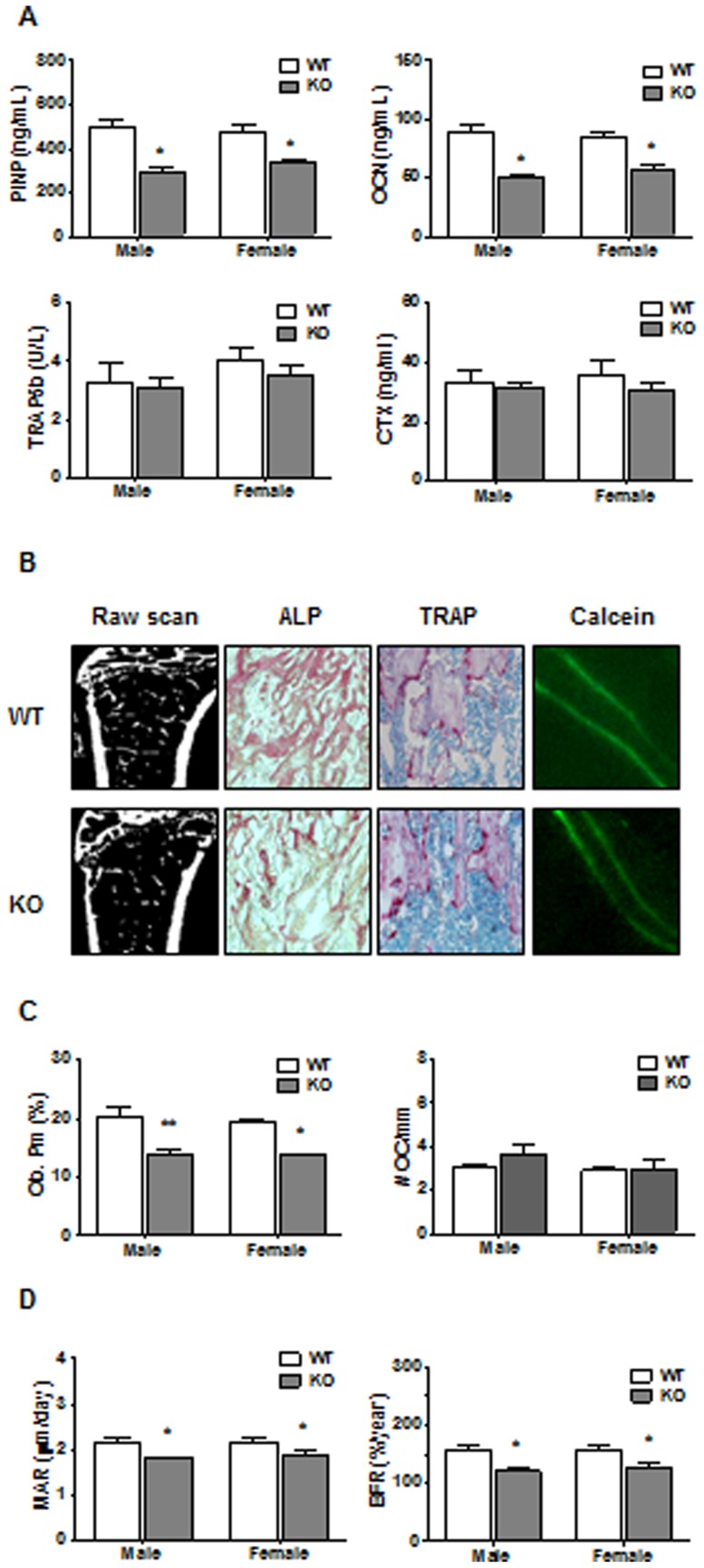
Plasma markers of bone remodeling and bone histochemical analysis of long bones from WT and CD36KO mice. A) Plasma levels of OCN, N-terminal propeptide of type I procollagen (PINP), tartrate-resistante acid phosphatase isoform 5b (TRAP5b) and C-terminal telopeptide of type I collagen (CTX) from 1 month-old male WT and CD36KO mice. Values are mean ± SEM from 3–9 mice. Student t test: **P<0.01 compared to WT mice. B–D) Raw scans, alkaline phosphatase (ALP), tartrate-resistant acid phosphatase (TRAP) and calcein stainings on representative bone sections from 1 month-old CD36KO and WT mice. Bone sections were used to evaluate relative osteoblast perimeter (Ob.Pm), number of osteoclasts (#Oc/mm) as well as mineral apposition rate (MAR) and bone formation rate (BFR). Values are means ±SEM from 3 mice in each group. Student t test: *P<0.05, **P<0.01 compared to WT mice

Since the low bone mass phenotype observed in CD36KO mice may result from an imbalance between the processes of bone resorption and formation, the plasma levels of bone remodeling markers were determined. As shown in Fig. 5A, levels of bone formation markers such as OCN and PINP were reduced in plasma of 1 month-old CD36KO mice compared to WT mice. On the other hand, levels of bone resorption markers, namely TRAP5b and CTX, were similar between CD36KO and WT mice. We further analyzed the bone tissue sections of CD36KO mice. As shown in Fig. 5B and C, lower relative ALP positive osteoblast perimeter were noticed in bone tissue of CD36KO mice compared to WT mice whereas numbers of TRAP positive osteoclast cells of bone tissue was similar between CD36KO and WT mice. Figure 5C shows representative calcein-stained interlabel distances in trabecular regions. As shown in Figure 5D, KO displayed reduced trabecular MAR and BFR.

### Functions of osteoblast from CD36KO mice

**Figure 6:**
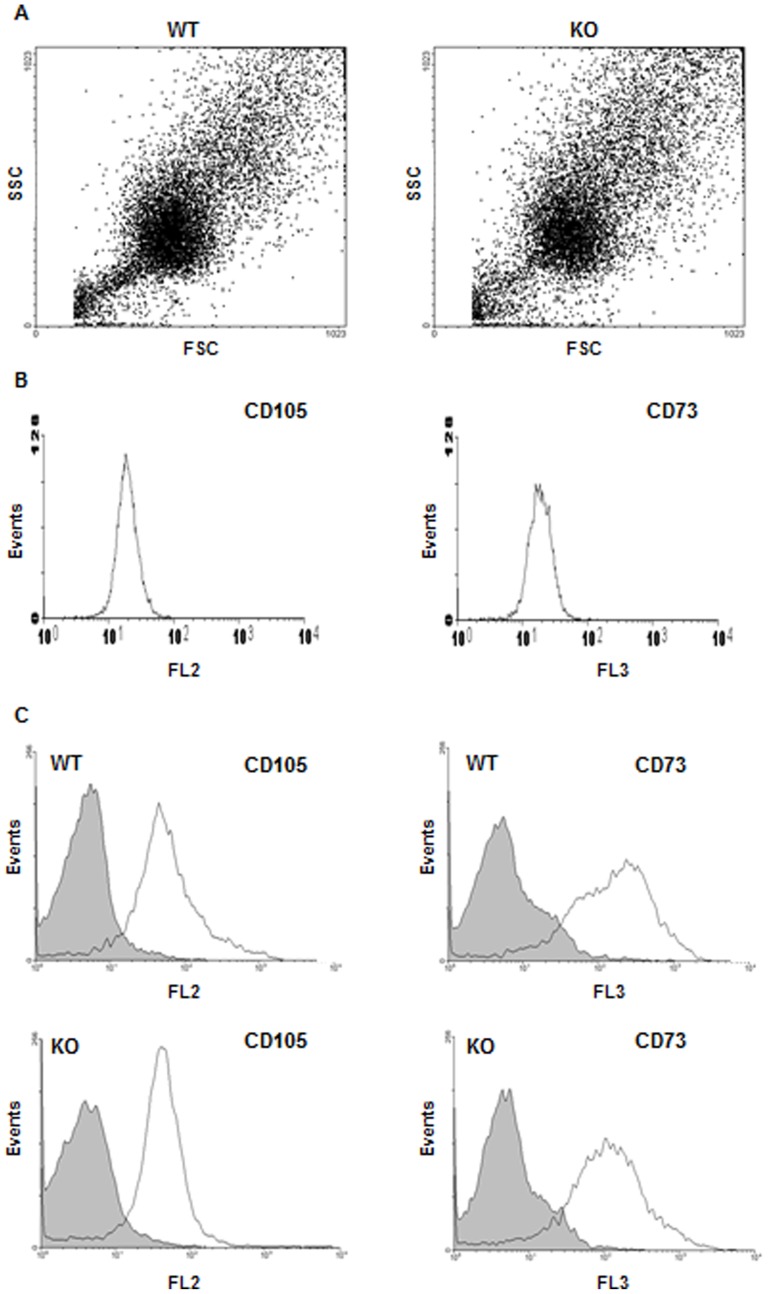
Phenotypic profile of bone marrow-derived MSC from WT and CD36KO mice. Flow cytometry analysis of MSC isolated from bone marrow of WT and CD36KO mice was performed on cells freshly isolated and adherent cells after 11 days of culture using CD105-PE and CD73-PerCP antibodies. A) Forward scatter (FSC) and side scatter (SSC) of the MSC population from bone marrow of WT and CD36KO mice at day 11 of culture. B) CD105-PE and CD73-FITC staining of the mesenchymal cell line C3H10T1/2. C) CD105-PE and CD73-FITC fluorescence for freshly isolated bone marrow cells (grey) and adherent cells after 11 days of culture (clear) from WT and CD36KO mice.

**Figure 7:**
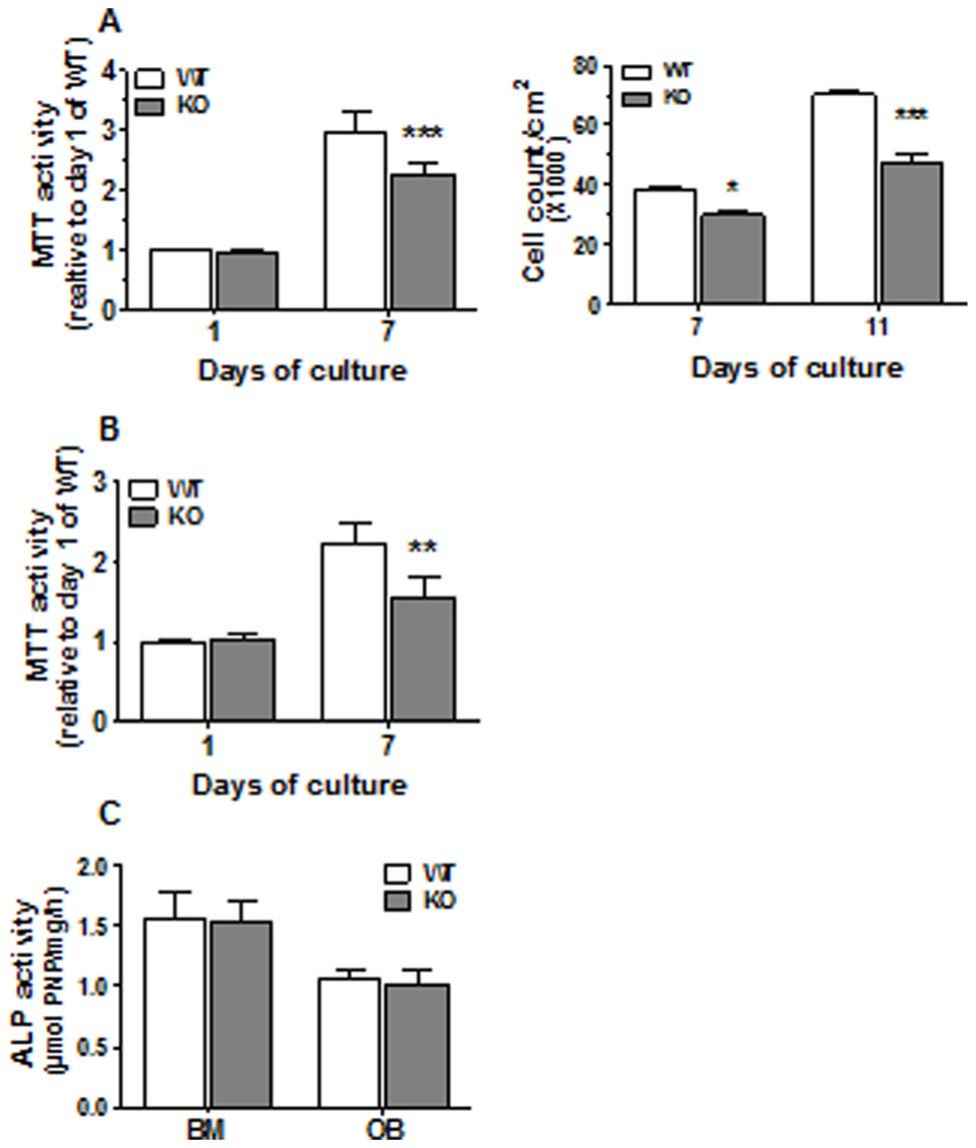
Cell culture expansion and alkaline phosphatase activity of CD36-deficient bone cells. MTT assay or cell counts was performed at day 1, 7 and 11 days post seeding in basal media for (A) MSC from bone marrow cells (BM) and (B) osteoblasts (OB) from bone fragments of WT and CD36KO mice. (C) Alkaline phosphatase activity was measured on MSC and OB after 7 days of culture. Data represent mean ± SEM of 5–9 independent experiments. Bonferroni post-test: **P<0.01, ***P<0.001.

Since histochemistry analyses and plasmatic levels of bone remodeling markers suggested potential dysfunction of bone formation process in CD36KO mice, we further evaluated the functions of MSC isolated from the bone marrow and osteoblasts from bone fragments of CD36KO mice. First, we analyzed populations of bone marrow derived MSC from WT and CD36KO mice by flow cytometry. At confluence, there were no differences for FSC and SSC parameters between MSC isolated from WT and CD36KO mice (Fig. 6A). Phenotypic characterization of MSC was carried out using cell surface markers CD105 and CD73. Validation of these mesenchymal markers was shown using the mouse mesenchymal cell line C3H10T1/2 with positive staining for CD105 and CD73 (Fig. 6B). As expected, at day of isolation, MSC positive for CD105^+^ and CD73^+^ account for minor fraction (<2%) of bone marrow cells from WT and CD36KO mice (Fig. 6C). Further culture of adherent cells for 11 days resulted in similar cell populations for WT and CD36KO as shown in Fig. 6A, which was positive (60–90%) for CD105 and CD73 (Fig. 6C). After confirming that primary cultures of MSC from WT and CD36KO mice were similar, we investigated cell culture expansion under basal culture conditions by MTT assays and cell counts. As shown in Fig. 7A, MTT activity at day 1 post-seeding was similar between cells from WT and CD36KO mice. However following 7 days of culture, the MTT activity of MSC from CD36KO marrow was lower by 24% than WT cells (Fig. 7A, left panel). In accordance with the reduction of MTT activity, reduced cell numbers were evidenced at 7 and 11 days of culture, being −22% and −32% respectively (Fig. 7A, right panel). Similarly, the MTT activity of osteoblasts from bone fragments of CD36KO mice was reduced by 30% when compared to WT cells (Fig. 7B). Although our results indicated reduced culture expansion of cells isolated from CD36KO mice when compared to WT mice, similar osteoblastic marker ALP activity was measured for cells from CD36KO and WT mice after 7 days of culture (Fig. 7C).

**Figure 8:**
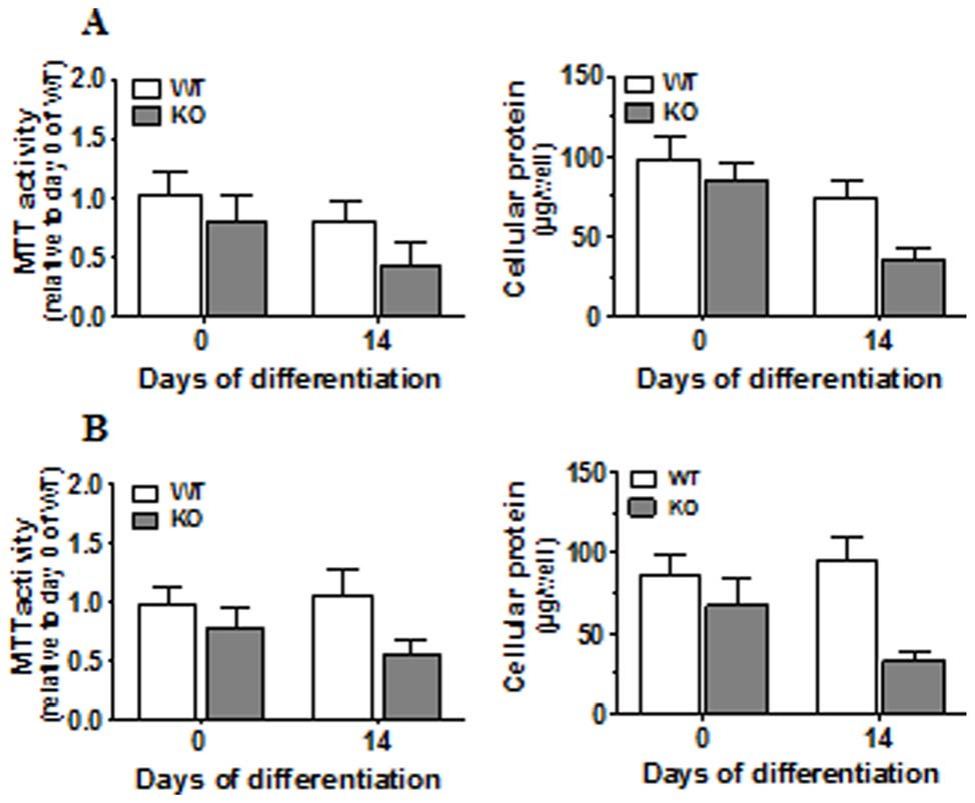
Cell Survival of CD36-deficient osteoblasts. MTT assays and protein measurements were performed on osteoblasts isolated from bone marrow (A) and long bone (B) of WT and CD36KO mice after 0 and 14 of culture in differentiation medium. Data represent mean ± SD of two independent experiments performed in triplicates.

The cellular lifespan of cells from CD36KO and WT mice was investigated under culture conditions with differentiation medium for 14 days. Differentiation treatments were initiated after 7 days of culture (designated day 0 of differentiation). Reduced culture expansion of CD36 deficient cells was again evidenced by lower cellular protein content and MTT activity of 13–24% at day 0 (Fig. 8). After 14 days of culture, cellular protein content and MTT activity further dropped to 37–53% in CD36-deficient MSC and osteoblasts from bone fragments (Fig. 8), suggesting that survival of cells lacking CD36 was impaired.

**Figure 9:**
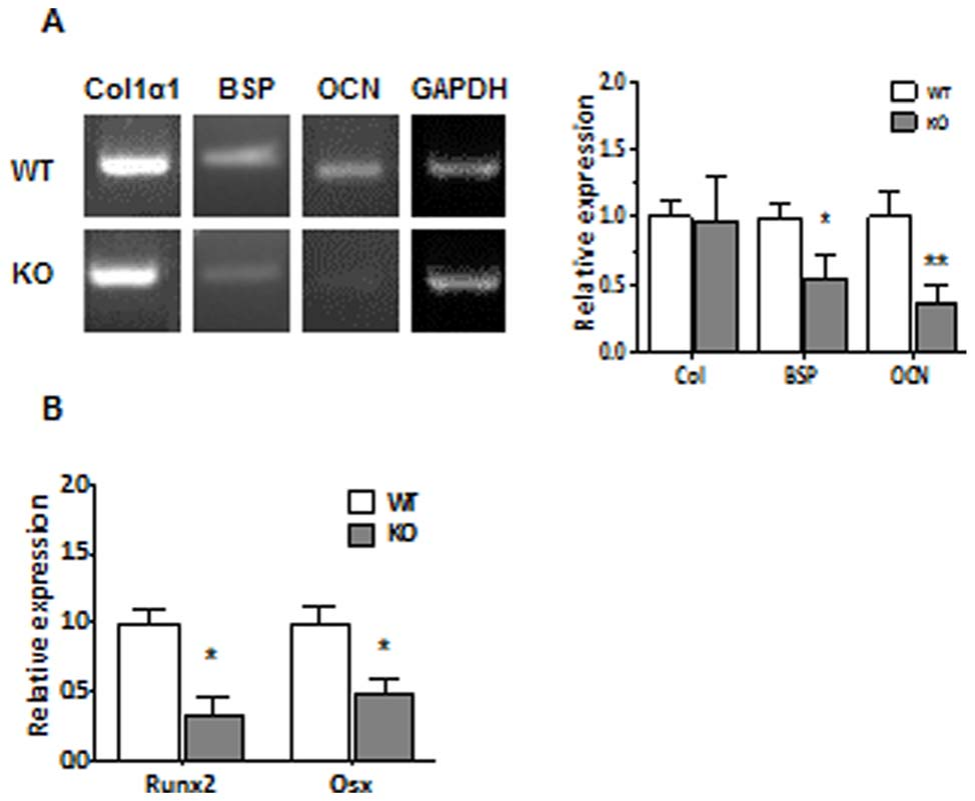
Expression of osteoblastic genes by cells from WT and CD36KO mice. Total RNA was isolated from MSC and cultured for 7 days. The levels of transcripts were determined by semi-quantitative RT-PCR using specific primers for (A) type I collagen (Col1α1), bone sialoprotein (BSP) and osteocalcin (OCN) or (B) osteoblastic transcription factors Runx2 and Osx as described in Material and methods. Expression levels were normalized against expression of reference gene. Data are mean ± SEM of 3–8 independent cell preparations. Student T test: *P<0.05, **P<0.01.

To further evaluate the mechanism leading to low bone mass phenotype in CD36KO mice, we determined the expression levels of osteoblastic genes. As shown in Fig. 9A, gene expression of Col1a1 in cells from CD36 KO mice was similar to cells from WT mice whereas gene expression of BSP and OCN was reduced. Moreover, PCR analysis showed that expression level of the osteoblastic transcription factors Runx2 and Osx was reduced in cells deficient for CD36 (Fig. 9B).

## Discussion

The principal aim of our study was to unravel the role of CD36 in bone metabolism and osteoblast functions. We report for the first time that CD36 ablation in mice leads to an osteopenic phenotype in trabecular bone. Low trabecular bone mass was observed in CD36KO mice at age of 1 month and was maintained up to 6 months for both genders. Femoral cortical bone mass of CD36KO mice tended to be lower although differences were not significant. Since changes in bone morphology are associated with imbalance between the resorption and formation processes, we determined plasma levels of bone remodeling markers. Plasma concentrations of bone formation markers, namely PINP and OCN, were diminished in 1 month-old CD36KO mice when compared to WT mice, whereas plasma levels of bone resorption markers were not altered. In accordance, histology analysis of bone sections highlighted lower numbers of ALP positive osteoblast cells. *In vitro* functional characterization of bone marrow-derived MSC and bone fragment-derived osteoblasts from CD36KO mice showed reduced cell culture expansion and survival. Moreover, gene expression of osteoblastic transcription factors Runx2 and Osx, as well as OCN and BSP was reduced in cells lacking CD36. Our results indicate that CD36 contributes to bone metabolism, playing a role in bone cell functions ensuring adequate bone formation.

As in comparison to WT mice, male and female CD36KOs showed lower weight from 1 to 6 months of age, we first asked if this lower weight reflected a reduction in bone growth. As reported, femoral and tibial lengths were similar between CD36KO and WT long bones indicating normal longitudinal bone growth of CD36KO mice. However, following dissection we noticed that visceral and fat pad adipose tissues were globally reduced in CD36KO mice compared to WT (unpublished observations) which may account for the reduced weight of CD36KO mice. In accordance with our observation, lower weight and decreased total body fat in male CD36KO mice have been previously reported [30]. To note, CD36 deficiency has been associated to impaired differentiation of adipocytes, adipocyte hypotrophy and reduction in their numbers in adipose tissue [31]. In another study, low weight of CD36KO mice was related to impaired CD36-mediated uptake of fatty acids to the peripheral, and particularly, to the adipose tissue [32]. Thus, decreased body weight in CD36KO animals may be a consequence of disturbance in the delivery of fatty acids and/or impaired adipogenesis. Low body weight is an established risk factor for low bone mass and fracture [33]. Given the involvement of CD36 in adipogenesis [31] and the reduced fat tissue and body weight of CD36KO mice, such low body weight may contribute to the reduced bone mass in CD36KO mice. To note, considering that leptin is primarily produced by white adipose tissue, and correlates positively with body fat, reduced fat tissue or lipoatrophy usually associates with low plasma levels of leptin [34]. Recently, it has been proposed that peripheral leptin has bone anabolic effects and contribute to bone formation [35].

Because of the role attributed to CD36 in the lipid metabolism and due to the possible association of lipid disorders and bone metabolism, we first determined the cholesterol and lipoprotein profiles of CD36KO mice. No significant difference was noticed in plasma levels of total cholesterol or cholesterol associated with lipoproteins between CD36KO and WT mice. Despite the large number of studies on lipid metabolism in CD36KO mice, the literature still reports contradictory results about blood levels of lipids. Several studies have shown increased plasma levels of fasting cholesterol [18], [36], non-esterified free fatty acid [18], [26], [32] and triglycerides in CD36KO mice [18], [32]. On the other hand, no differences for some of these parameters have also been reported in mice lacking CD36 [26], [32], [36]. Also, reduced levels of total cholesterol have been measured in male CD36KO mice [26]. We observed similar reduction of total cholesterol in male CD36KO mice although this difference was not significant. Therefore, the absence of alteration in plasma cholesterol levels of CD36KO mice observed in our study is not much surprising and such discrepancy has generally been attributed to the starving period or the method of blood collection. Secondly, we analysed general plasmatic parameters related to bone metabolism. Plasma levels of calcium, phosphate and alkaline phosphatase were similar between CD36KO and WT mice which indicate that global mineral homeostasis is not altered in these mice.

To determine the role of CD36 in bone metabolism, we undertook the analysis of the bone microarchitecture of CD36KO mice. The bone volume of femoral trabecular portion and of vertebrae was reduced in 1 to 6 month-old CD36KO mice of both genders. Of interest, we observed a lack of increase in trabecular bone volume with age in CD36KO mice which points out some impairment in bone formation. Similarly, global lower bone mass by 12% was reported in male CD36KO mice by Hajri et al. [30]. The lower bone mass in CD36KO mice is explained by the diminution of trabeculae number which translates in greater trabecular separation. However no significant difference was noticed for the cortical portion of femura although tendency to reduced bone mass was observed. It is generally assumed that the mechanisms and rate of bone remodeling are different in trabecular *versus* cortical bone [37], the former showing faster responses to metabolic changes [38] with higher rate of bone remodeling. Therefore, the endochondral bone modeling seems globally normal in CD36KO mice although bone remodeling at the trabecular portion may be altered. Since both gender of CD36KO mice showed a low bone mass phenotype, dysfunction of bone remodeling in CD36KO mice does not appear to be related to sex steroid status.

Multiple mechanisms may be responsible for decreased bone mass. Independently of the cause of bone metabolism disruption, loss of bone tissue originates from an imbalance between the processes of bone resorption and formation which results from altered regulation/functions of bone cells. The levels of the bone formation markers PINP and OCN were reduced in CD36KO mice whereas the levels of bone resorption markers, namely CTX and TRAP5b, were not altered. In accordance with a dysfunction of bone formation, numbers of ALP positive cells were reduced in bone tissue sections from CD36KO mice (Fig. 3B) whereas similar number of osteoclasts was evidenced in WT and CD36KO mice. Therefore, both plasma levels of bone remodeling markers and bone histomorphometric analysis point to an impaired bone formation in CD36KO mice. Given our results which indicate similar plasma levels of bone resorption markers and number of TRAP positive osteoclasts in bone sections, cell function/differentiation of osteoclasts from CD36 KO mice was not further investigated. CD36 has been associated to cytokine-induced macrophage fusion into multinucleated giant cells [39]. Despite that macrophage fusion leads also to osteoclast formation, Helming et al. [39] noticed that osteoclast formation was not altered in the absence of CD36, suggesting its selective involvement in cytokine-induced macrophage fusion for giant-cell formation. Moreover, impaired fusion of osteoclasts from KO-CD36 bone marrow progenitors would lead to lower bone resorption, being inconsistent with the low bone mass revealed in CD36KO mice.

Since our results indicate that the osteopenic phenotype in CD36KO mice could be due to impaired osteoblast-mediated bone formation, we further investigated the functions of cells isolated from bone marrow and bone fragments of CD36KO mice. Our data showed a decrease culture expansion potential (24–30%) of cells lacking CD36, either isolated from the bone marrow as MSC and from the bone fragments as osteoblasts. Nevertheless, bone cells from WT and CD36KO mice showed comparable ALP activities suggesting that cell preparations from WT and CD36KO mice were similar in terms of the cell populations, which was confirmed by phenotypic analysis. The involvement of CD36 in cell proliferation has been reported previously. CD36 deficiency has been shown to reduce the proliferation of astrocytes and delayed closure of the wound gap [40]. Moreover, ribozyme-mediated down regulation of CD36 was shown to inhibit growth of thrombospondine 1 (TSP1)-expressing cells [41]. Interestingly, our results indicate that cell survival under osteogenic culture conditions was reduced in cells deficient for CD36. Also, as cell apoptosis was not investigated, we cannot exclude that cells from CD36KO mice are more sensitive to apoptosis. Such impaired osteoblastic bone formation caused by decreased number and activity of individual osteoblastic cells was shown to be responsible of reduction of bone mass in male patients with idiopathic osteoporosis [42].

To further investigate the functions of cells lacking CD36, we determined the expression levels of key genes of osteoblast functions [1], [2]. Our results indicated that bone marrow-derived MSC from CD36KO mice have low gene expression of Runx2, Osx, OCN and BSP when compared to WT mice whereas Col1α1 gene expression was similar. Such impairment in osteoblastic gene expression further suggests that bone cells functions are altered in CD36KO mice. Given that we evidenced altered cell survival of CD36-deficient cells, reduced Von Kossa and Alizarin red stainings that was observed (data not shown) likely arise from lower density in culture dishes of cells from CD36KO mice. Therefore, further experiments are needed to reveal mechanistic dysfunctions in CD36-deficient cells and such functional impairment are currently under investigation.

In conclusion, our results show that CD36KO mice show an osteopenic phenotype which correlates with impaired bone formation and altered functions of osteoblasts. Further studies are warranted to document mechanisms that link CD36 deficiency to osteoblast dysfunctions.
